# Longitudinal Effects of Embryonic Exposure to Cocaine on Morphology, Cardiovascular Physiology, and Behavior in Zebrafish

**DOI:** 10.3390/ijms17060847

**Published:** 2016-05-31

**Authors:** Eric J. Mersereau, Cody A. Boyle, Shelby Poitra, Ana Espinoza, Joclyn Seiler, Robert Longie, Lisa Delvo, Megan Szarkowski, Joshua Maliske, Sarah Chalmers, Diane C. Darland, Tristan Darland

**Affiliations:** 1 Biology Department, University of North Dakota, 10 Cornell Street, Grand Forks, ND 58202, USA; emersereau93@midwestern.edu (E.J.M.); cody.boyle.1@und.edu (C.A.B.); Shelby.poitra@gmail.com (S.P.); joclyn.seiler.2@my.und.edu (J.S.); Robert.a.longie@gmail.com (R.L.); lisadelvo@gmail.com (L.D.); meganszarkowski@gmail.com (M.S.); josh.k.maliske@gmail.com (J.M.); Sarah.J.Chalmers@osfhealthcare.org (S.C.); diane.darland@und.edu (D.C.D.); 2 Department of Ecology and Evolutionary Biology, University of Arizona, Tucson, AZ 85721, USA; amespinoza@email.arizona.edu

**Keywords:** cocaine, zebrafish, cardiovascular physiology, and behavior

## Abstract

A sizeable portion of the societal drain from cocaine abuse results from the complications of *in utero* drug exposure. Because of challenges in using humans and mammalian model organisms as test subjects, much debate remains about the impact of *in utero* cocaine exposure. Zebrafish offer a number of advantages as a model in longitudinal toxicology studies and are quite sensitive physiologically and behaviorally to cocaine. In this study, we have used zebrafish to model the effects of embryonic pre-exposure to cocaine on development and on subsequent cardiovascular physiology and cocaine-induced conditioned place preference (CPP) in longitudinal adults. Larval fish showed a progressive decrease in telencephalic size with increased doses of cocaine. These treated larvae also showed a dose dependent response in heart rate that persisted 24 h after drug cessation. Embryonic cocaine exposure had little effect on overall health of longitudinal adults, but subtle changes in cardiovascular physiology were seen including decreased sensitivity to isoproterenol and increased sensitivity to cocaine. These longitudinal adult fish also showed an embryonic dose-dependent change in CPP behavior, suggesting an increased sensitivity. These studies clearly show that pre-exposure during embryonic development affects subsequent cocaine sensitivity in longitudinal adults.

## 1. Introduction

The National Institute on Drug Abuse recently reported that as many as 855,000 Americans were abusing cocaine in 2013 [1] and that the estimated cost to society approached $193 billion dollars in 2007 and 2008 [2]. Further costs were incurred because of developmental difficulties in children potentially caused by cocaine exposure during pregnancy. In 2008 and 2009, the number of women using cocaine during pregnancy ranged between 2% in women aged 26–44 to a staggering 15.8% in women aged 15–17 [3]. Lower birth weight and smaller brain size have been reported for newborns exposed to cocaine *in utero* [4,5,6]. Cardiovascular abnormalities [7,8], cognitive difficulties, and subsequent behavioral issues, including an increased likelihood for substance abuse, have all been reported for adolescent children exposed to cocaine *in utero* (recently reviewed by [9,10]). The data on the effects of cocaine *in utero* are confounded by other variables including false reporting, ingestion of other substances and the nutritional state of the mother. However, many of these findings have been verified using model organisms, principally rodents.

Use of model organisms allows carefully controlled administration of the cocaine to pregnant mothers and detailed analysis of cellular and molecular abnormalities associated with *in utero* drug exposure [11,12,13,14]. Decreased brain size has been demonstrated in rodents and monkeys treated with cocaine *in utero* [11,14,15,16]. Mice raised after *in utero* cocaine exposure have behavioral deficits [12] and acquire cocaine self-administration more quickly than untreated controls [17]. The nutritional state of the mother, restricted or differential perfusion of the embryos by the placenta, and limitations on the window of drug exposure in rodents are still potential confounds of using mice. Other model organisms might provide additional insights as to the impact of cocaine exposure on brain and heart development and the longitudinal effects of that exposure.

The zebrafish (*Danio rerio*) offers some advantages over mammals in longitudinal toxicology studies in that development is external, leaving no questions about the impact of the nutritional state of the mother or embryonic blood supply during development. The zebrafish embryos are transparent and can be analyzed noninvasively and allowed to mature to adulthood. For these reasons and others, the zebrafish has become more popular as a model organism for examining human neuropsychiatric disorders in recent years (for recent reviews, see [18,19,20]). While the earliest phases of development are essentially conserved between the teleosts and mammals, forebrain development in zebrafish is somewhat different. Development of the telencephalon in teleosts involves an eversion, rather than evagination, resulting in a rearrangement of the ventricles and sub regions relative to mammals [21,22,23,24]. Despite these anatomical differences, functional conservation remains. A region identified as the amygdala, using specific molecular markers, also mediates fear, anxiety and avoidance in zebrafish, much as it does in mammals [21,25,26]. The reward circuitry in teleosts is also similar in basic anatomy to that in other vertebrates [24]. Zebrafish are behaviorally responsive to cocaine as adults and the telencephalon, which includes the striatum, is certainly involved in the response to cocaine [27,28]. This suggests that the zebrafish would make an excellent model to study the effects of cocaine on embryonic development and the longitudinal effects on cardiovascular physiology and drug-related behavior. We show that embryonic cocaine exposure increases physiological and behavioral sensitivity later in adulthood.

## 2. Results

### 2.1. Cocaine Dosage Effects on Embryonic Health and Brain Development

**Figure 1:**
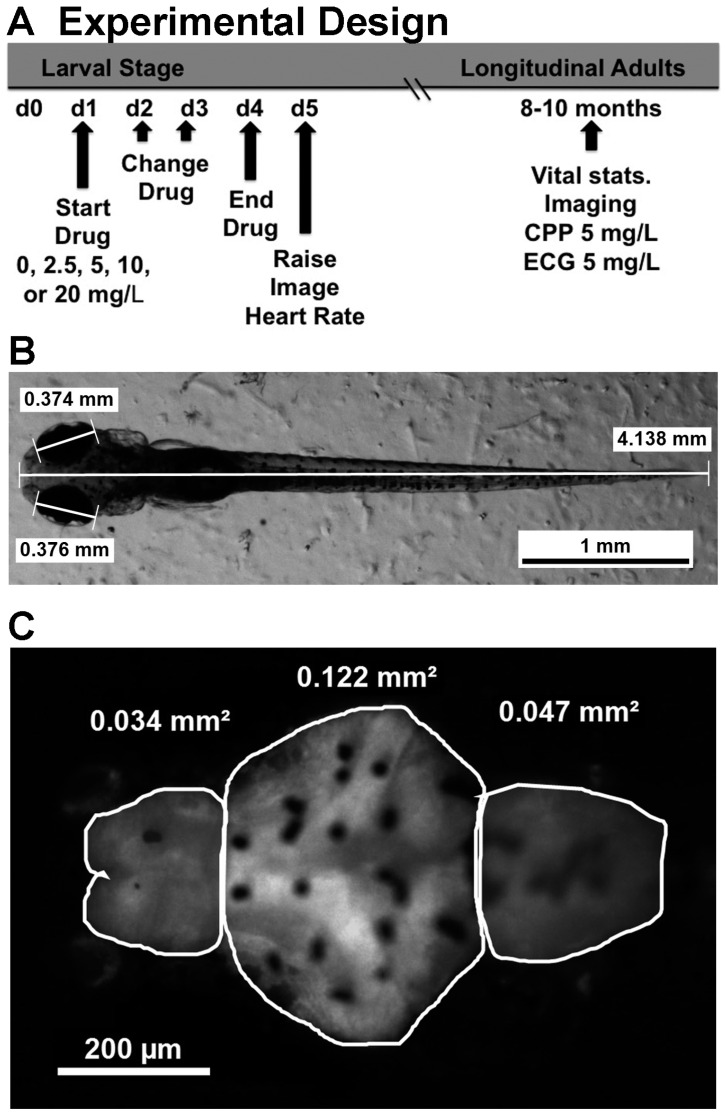
Experimental design of cocaine treatment and analysis of five-day larval zebrafish. A schematic showing the time course of embryonic drug exposure, imaging and longitudinal analysis is shown in (**A**); (**B**) shows an example of body length and eye diameter measurements made under bright field illumination; (**C**) shows the same fish under fluorescence illumination, focusing specifically on the brain at higher magnification. The tracing outlines the telencephalon (Tel), the diencephalon (Dien, which actually includes the optic tectum, midbrain and cerebellum), and the hindbrain (Hind, which includes the rhombencephalon), with sample area measurements given for each region.

Fish larvae were treated with cocaine as described in the methods section and Figure 1. Several parameters were examined to assess overall health of embryonic zebrafish treated with different doses of cocaine (Table 1). One obvious effect of the cocaine treatment was accelerated hatching. From 12 experiments, in which we compared fish treated with different doses of cocaine (the minimum number of experiments for each dose was six), there was a clear cocaine dose-dependent increase in hatching at 48 hpf (Table 1). Fish treated with 5, 10 and 20 mg/L had a significantly higher proportion of hatched fish than untreated controls at the same time point (*p* < 0.05, *p* < 0.01, and *p* < 0.001, respectively). Despite the earlier hatching, there was no significant change in body size detected between treatment groups (Table 1, *p* < 0.96, *F* = 0.145, df = 214), indicating that overall body growth was not affected by cocaine. Similarly, eye size was not statistically different between control and treatment groups (Table 1, *p* < 0.12, *F* = 1.834, df = 221).

**Figure 2:**
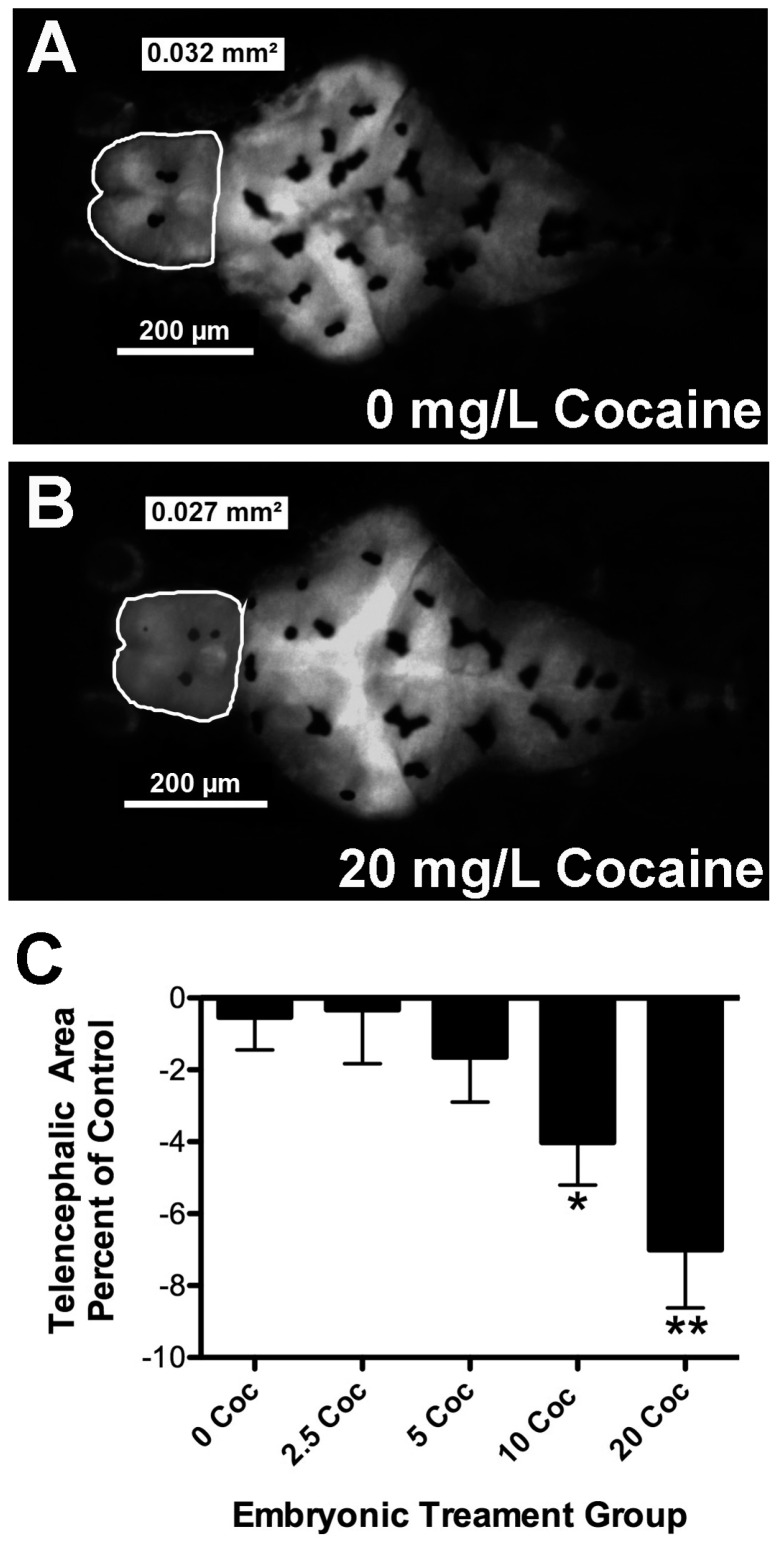
Cocaine treatment decreases telencephalic area of zebrafish larvae. There was a readily observed decrease in telencephalic size seen after treatment with the highest doses of cocaine. Panels A and B provide a comparison between untreated fish (**A**) and siblings treated with 20 mg/L cocaine (**B**); In (**C**), results from several experiments were combined, with brain size expressed as a percentage of untreated controls. Brain size was reduced on average 7% by treatment with 20 mg/L cocaine. Error bars signify ± SEM, * *p* < 0.05 relative to control and ** *p* < 0.01 relative to untreated control.

To determine if cocaine affects gross brain morphology in zebrafish, brain size was determined by imaging the brains of five-day GFP^+^ larval fish treated with different doses of cocaine. There was a clear difference in telencephalic area observed in five-day-old embryos treated with 10 and 20 mg/L of cocaine. Image analysis of embryonic brains confirmed that cocaine exposure resulted in decreased telencephalic area in a dose-dependent fashion (Table 1, Figure 2). If compared as a percentage of untreated controls, 10 and 20 mg/L cocaine caused significant decreases in telencephalic size (Figure 2C, * *p* < 0.05, and ** *p* < 0.01 respectively, *F* = 3.71, df = 110), with a maximal effect at 20 mg/L of about 7%. There was no statistical difference between treatment groups in the diencephalon or the hindbrain area (Table 1: *p* < 0.145, *F* =1.745, df =122 and *p* < 0.149, *F* = 1.727 and df = 114 respectively). In preliminary experiments, we compared chorionated embryos with dechorionated embryos treated with 0 and 20 mg/L cocaine (data not shown). Chorionated embryos and dechorionated embryos showed nearly identical decreases in telencephalic area, suggesting that cocaine readily passes through the embryonic membrane (data not shown). AO staining was performed to determine if the difference in telencephalic size was due to neurotoxicity and subsequent cell death caused by early drug exposure. Numbers of AO-positive (AO^+^) cells did not vary significantly between embryonic treatment groups (Table 1: *p* < 0.15, *F* = 1.52, df = 109).

**Figure 3:**
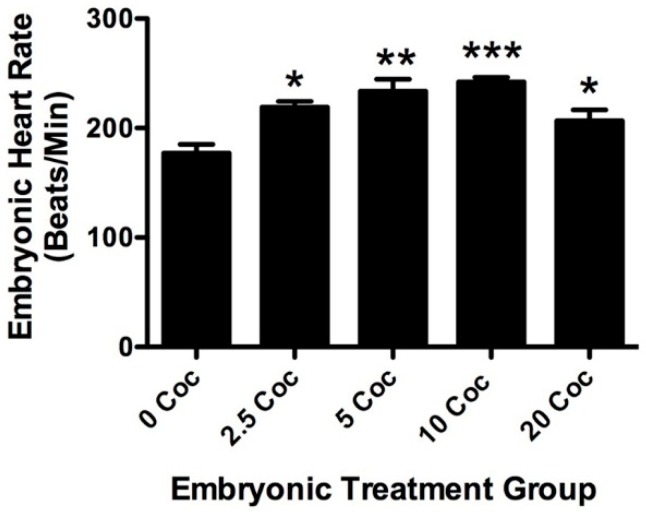
Cocaine treatment during development alters heart rate in zebrafish larvae. The histogram shows data from one of three experiments measuring heart rate in zebrafish larvae exposed to different doses of cocaine for three days and then allowed one day of recovery before testing. Cocaine induced a bell-shaped dose response curve in larval baseline heart rate, with the maximal effect seen after treatment with 10 mg/L (error bars indicate ± SEM, * indicates *p* < 0.05, ** indicates *p* < 0.01, *** indicates *p* < 0.001 compared to untreated controls, *n* = 8 for each condition).

In addition to body size and brain morphology, heart rate after three days of exposure to cocaine and one day of recovery was assessed in zebrafish larvae. The average heart rate of untreated fish was 177 ± 8 beats per minute, similar to what we have previously reported [29]. Figure 3 shows one of three experiments, all with similar results, looking at the effects of three days treatment at different doses of cocaine, followed by one day of recovery without drugs. There is a bell-shaped response curve to cocaine among the treatment groups, but all treatments resulted in significantly higher baseline heart rates than in untreated control larvae (*p* < 0.05 for 2.5 mg/L, *p* < 0.01 for 5 mg/L, *p* < 0.001 for 10 mg/L, and *p* < 0.05 for 20 mg/L). The most effective dose was 10 mg/L, which elevated heart rate 37% above untreated fish. This dosage was also significantly higher than that achieved with 20 mg/L (*p* < 0.05). To see if changes in heart rate were linked to cytotoxicity, fish were examined for AO staining in the heart muscle. There were very few AO^+^ cells detected in any of the treatment groups and there was no statistical difference between them (data not shown, *p* < 0.66, *F* = 0.245, df = 40). While the subtleties of heart morphology could not be assessed in these experiments due to different angles and low resolution of imaging, gross morphology was normal in all larvae examined (the ventricle was always on the left side). Videos were reviewed for abnormalities in heart rhythm. In 120 recordings, 11 fish were found with apparent arrhythmias. Four of these arrhythmias were found in untreated fish, three were found in fish treated with 2.5 mg/L, two in fish treated with 5 mg/L, one in fish treated with 10 mg/L and one in fish treated with 20 mg/L cocaine. Nine of these arrhythmias involved intermittent increases in ventricular volume followed by longer and slower contraction. The other two showed an obvious skip in heartbeat.

### 2.2. Longitudinal Effects of Cocaine on Zebrafish Health, Cardiovascular Physiology and Behavior

We considered several parameters in assessing the effects of early cocaine exposure on overall health of longitudinal adult fish (Table 2). Embryonic cocaine treatment had no effect on survival after 8–10 months (*p* < 0.98, *F* = 0.08, df = 28). Sex ratios of clutches were also unaffected (*p* < 0.55, *F* = 0.775, df = 34). Similarly, body length and body weight did not differ between the treatment groups (*p* < 0.205, *F* = 1.52, df = 132, and *p* < 0.42, *F* = 0.98, df = 132 respectively). When all treatment groups were considered together, females weighed significantly more than males (0.47 ± 0.1 and 0.41 ± 0.09 g, *p* < 0.001) and were also significantly longer than males (3.85 ± 0.26 and 3.58 ± 0.23 cm *p* < 0.0001). However, embryonic cocaine dose did not significantly affect body length or weight in either sex (data not shown, for male body length *p* < 0.178, *F* = 1.66, df = 56, for male body weight, *p* < 0.099, *F* = 2.059, df = 68, for female body length *p* < 0.174, *F* = 1.70, df = 66, and for female body weight, *p* < 0.26, *F* = 1.25, df = 84). The telencephalic area was imaged under GFP optics and used to assess brain size. There was a general trend towards increased telencephalic area with dose, but this was not statistically significant (Table 2, *p* < 0.196, *F* = 1.55, df = 84). Again, this was true regardless of whether the sexes were considered separately (for males *p* < 0.63, *F* = 0.65, df = 42, and for females *p* < 0.76, *F* = 0.42, df = 38). Relative thickness of the skull and cranial pigment obscured visualization with this approach such that adult measurements on the diencephalon and hindbrain were not collected.

**Figure 4:**
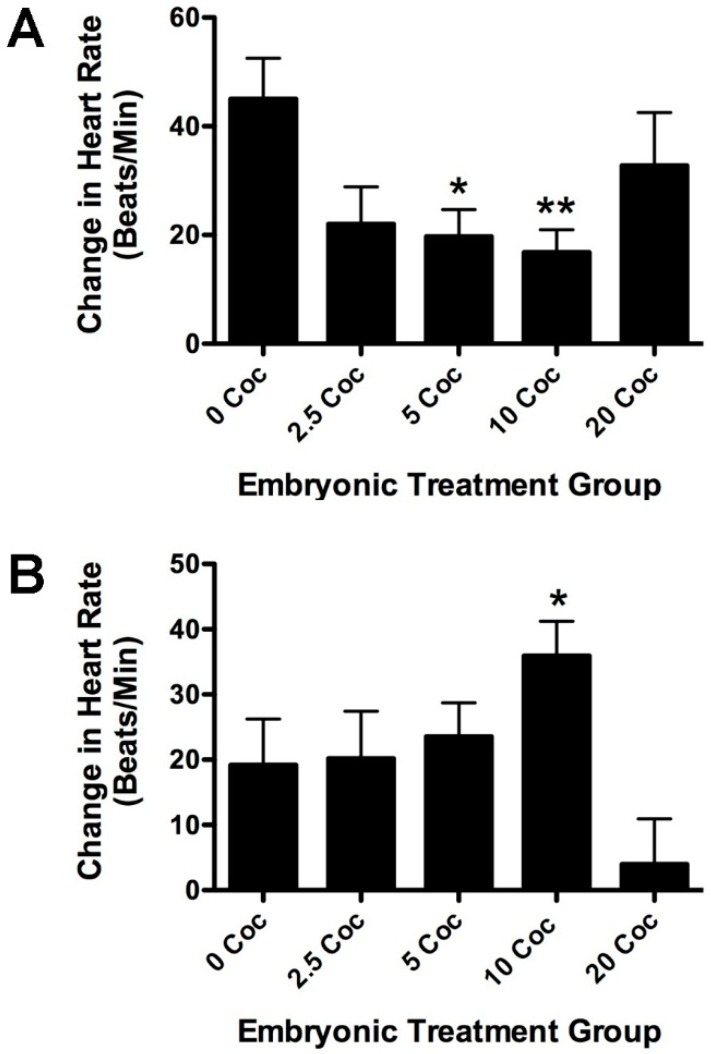
Embryonic cocaine exposure alters cardiovascular sensitivity to isoproterenol (**A**) and cocaine (**B**) in longitudinal adults. The heart rate of longitudinal adults was determined by measuring the ECGs. Baseline heart rate in longitudinal adults treated with cocaine as larvae was no different from untreated controls (Table 2). However, the response to isoproterenol, a ß1 agonist, was lower in longitudinal fish that were treated with cocaine as larvae than in fish that did not receive cocaine. The histogram in panel A shows the isoproterenol-induced change from baseline heart rate in one of three experiments all showing similar results. The effect of larval pre-exposure to cocaine on the adult isoproterenol response was U-shaped, with a maximal inhibition seen in fish previously treated with 10 mg/L cocaine (Error bars indicate ± SEM, * indicates *p* < 0.05, ** indicates *p* < 0.01, compared to untreated controls, *n* = 7 for each condition). Longitudinal fish from the different embryonic treatment groups were also challenged with 5 mg/L cocaine and their ECGs measured. The histogram in panel B shows the effect of larval pre-exposure to cocaine on subsequent adult cardiovascular response to the drug. The response by the different longitudinal groups is bell-shaped, with a maximal effect seen in fish pre-exposed to 10 mg/L cocaine as larvae (Bars indicate ± SEM, * indicates *p* < 0.01 when compared to fish pre-exposed to 20 mg/L, *n* = 18 for 0 and 20 mg/L cocaine, *n* = 16 for 2.5 and 10 mg/L, and *n* = 15 for 5 mg/L cocaine).

ECGs were recorded to see how cardiovascular activity differed between longitudinal groups (Table 2 and Figure 4A,B). Baseline heart rates for anesthetized fish were similar to what we have previously reported [29]. There was no significant difference in baseline heart rate between adults raised from the embryonic treatment groups (Table 2, *p* < 0.77, *F* = 0.452, df = 60). In order to gauge the cardiovascular response to stress, heart rate was measured after exposure to 20 µM isoproterenol, a potent ß1-adrenergic agonist (Figure 4A). In previous studies, this dose proved very effective at increasing the heart rate [29]. In control fish, which had not been exposed to cocaine as embryos, isoproterenol significantly increased heart rate by 32% (repeated measures ANOVA, *p* < 0.003). Isoproterenol also increased heart rate significantly in the other embryonic treatment groups (for 2.5 mg/L, *p* < 0.003; for 5 mg/L, *p* < 0.0007; for 10 mg/L, *p* < 0.006; for 20 mg/L, *p* < 0.02). However, the response of the 5 and 10 mg/L groups were significantly lower than the response of control fish not treated with cocaine as embryos (*p* < 0.05 and *p* < 0.01, respectively). In contrast, fish treated with 20 mg/L cocaine as embryos, had an isoproterenol response similar to the untreated longitudinal control group. Thus, in longitudinal adults, a U-shaped dose response curve to isoproterenol was seen that mirrored the bell-shaped response curve of baseline heart rate at 5 dpf. The adult fish were also treated with an optimal dose of cocaine as previously determined (5 mg/L, [29]) to test if embryonic exposure to the drug sensitized cardiovascular response later (Figure 4B). The cardiovascular cocaine response of the embryonic treatment groups was provocatively bell-shaped; however, the differences between the treated groups and untreated controls were not quite statistically significant (*p* < 0.06, *F* = 2.37, df = 92).

In 93 recordings assessed for arrhythmia, only five were identified as such by more than one investigator. Three of these were from fish previously exposed to 10 mg/L cocaine. The other two recordings with possible arrhythmias were taken from one fish previously exposed to 2.5 mg/L and one previously exposed to 20 mg/L. Two of these displayed an occasional extra P-wave, while the others had an apparent skip in heartbeat. These effects were quite subtle and overall cocaine pre-exposure had very little effect on heart rhythm in longitudinal adults.

**Figure 5:**
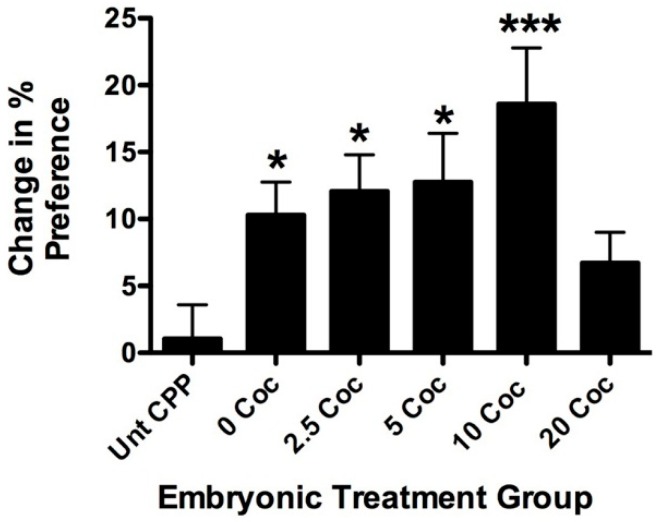
Pre-exposure to cocaine in larval fish affects cocaine-induced conditioned place preference (CPP) in longitudinal adults. This histogram represents data from four experiments combined testing CPP in longitudinal adults raised from larvae exposed to different doses of cocaine. Fish from all embryonic treatment groups not treated with cocaine as adults were combined into a single group for comparative purposes (Unt CPP). CPP was tested using 5 mg/L for all embryonic treatment groups and is expressed as a change in the percentage of time spent in the conditioning chamber before and after drug exposure. Cocaine pre-exposure during development results in a bell-shaped response curve for CPP in longitudinal adults. All longitudinal groups showed significantly higher CPP than untreated controls except for fish previously exposed to 20 mg/L cocaine. The maximal response observed in fish previously exposed to 10 mg/L and this group had the lowest *p*-value and was also significantly higher than the 20 mg/L group (*p* < 0.05). (Error bars represent ± SEM, * indicates *p* < 0.05, *** indicates *p* < 0.001 when compared to the untreated control group, *n* = 33 for untreated controls, *n* = 44 for 0 mg/L cocaine, *n* = 22 for 2.5 mg/L, *n* = 22 for 5 mg/L, *n* =30 for 10 mg/L, and *n* = 37 for 20 mg/L).

To test whether developmental exposure to cocaine affects behavioral sensitivity with secondary exposure later, longitudinal adult fish from the five embryonic treatment groups were tested for cocaine-induced CPP (Figure 5). All fish were tested with 5 mg/L cocaine, a dose shown to be submaximal in previous studies [27,28]. A subset from each treatment group was tested for CPP without cocaine. These untreated controls showed no significant variance in CPP based on embryonic treatment (data not shown *p* < 0.35, *F* = 1.24, df = 24) and were, therefore, combined in Figure 4 for statistical comparison (Unt CPP in Figure 5). The 0 mg/L (*p* < 0.02), 2.5 mg/L (*p* < 0.03), 5 mg/L (*p* < 0.01), and 10 mg/L (*p* < 0.001) embryonic treatment groups all displayed significant CPP compared to baseline readings. In contrast, Unt CPP fish did not show significant CPP (*p* < 0.28). Interestingly, fish raised after embryonic treatment with 20 mg/L did not show significant CPP. The dose response for the embryonic treatment groups is bell-shaped with maximum CPP observed in the fish treated with 10 mg/L during development. This was not only the highest value above the Unt CPP fish (*p* < 0.001), but also significantly higher than fish treated with 20 mg/L cocaine as embryos (*p* < 0.02).

## 3. Discussion

In this study, we show that embryonic cocaine exposure sensitizes cardiovascular and behavioral response to the drug in longitudinal adult zebrafish. The mechanism driving sensitization is still unknown. Cocaine exerts its physiological effects in two ways. First, it blocks monoaminergic transporters, thereby raising synaptic levels of dopamine, serotonin and catecholamines [30,31]. Second, unlike many other monoamine reuptake inhibitors, cocaine also inhibits voltage-gated ion channels at lower potency [32]. The rewarding aspects of drug use, which have been linked to their addictive potential, are mediated by the increased monoamine neurotransmission, particularly of dopamine and serotonin [33,34]. Cocaine has biphasic effects on heart rate [35,36]. Cocaine increases heart rate at low doses by effects on catecholaminergic peripheral synapses and on central sympathetic output pathways. The inhibitory effects on ion channels at higher concentrations produce many of the deleterious effects on heart rate and cardiovascular rhythm. The primary developmental effects of cocaine are believed due to elevated monoamines, principally dopamine. This is largely due to dopamine’s well-documented effects on neural development [37,38,39,40].

The earliest observed effect of cocaine in the present study was a dose-dependent precocious hatching by 48 hpf. Others have observed changes in gene expression in zebrafish larvae treated with cocaine, which might underlie development of the peripheral nerves and body muscle chevrons [41]. Perhaps this is the basis for earlier hatching, although cocaine dose did not appear to speed development in other respects such as body or brain size. Alternatively, precocious hatching could represent a stress response to a suboptimal environment.

Cocaine’s effects on embryonic morphology were very mild, except for a readily observable dose-dependent decrease in telencephalic area at higher doses. The fact that preliminary experiments showed no impact of dechorionation on telencephalic size decrease and that there was an obvious difference in hatching time, suggests that cocaine readily penetrates the chorion. The cause for the decrease in telencephalic area with cocaine treatment is unknown, but the AO staining experiments suggest that cytotoxicity is unlikely. This was in marked contrast to parallel experiments using heavy metals in our laboratory, which show a dose-dependent increase in AO staining (data not shown). General toxicity also seems unlikely because hindbrain and diencephalon size were unaffected by cocaine treatment, despite sending and receiving extensive monoaminergic input [42,43,44,45]. In rodents and primates treated with cocaine *in utero*, there is a common finding of decreased cortical volume and delayed, or decreased lamination of the cortex [11,16]. The authors suggested that rather than altered neurogenesis, it is survival, differentiation, and migration of neuronal precursors that are affected by cocaine. In addition to delays in radial migration of cells from the ventricular proliferative zone, altered tangential migration of subpallial GABAergic precursor cells into the developing cortex contributes significantly to the lamination defects seen [11,13,15]. Although teleosts lack a neocortex, comparable developmental pathways occur in the teleost telencephalon. Proliferative cells in the ventricular zone migrate radially, giving rise to the glutamatergic cells of the pallium that will eventually become the hippocampus, amygdala, isocortex and the olfactory sensory nuclei [22,23]. In addition, Dlx2, Lhx7 and GAD67-positive neuronal precursors arising from the subpallium migrate into the dorsal-lateral pallium to become GABAergic interneurons [23]. The decrease in telencephalic area seen in cocaine-treated zebrafish embryos might therefore be due to slower expansion of intracellular connections and neuropil formation in the pallium. Current, ongoing studies looking at marker expression will elucidate whether altered proliferation, differentiation, or migration is mediating the effects of cocaine on telencephalic size in zebrafish. A related question is whether decreased telencephalon size impacts performance in the CPP test. The dorsal nucleus of the ventral telencephalic area (Vd) is believed to include the teleost equivalent of the nucleus accumbens [24], and is a region where gene expression changes induced by cocaine are particularly evident [28]. Marker expression studies of the developing embryo described above combined with studies for Vd markers like substance P and the D1 dopamine receptor [24] in longitudinal adults should determine if particular cell groups are affected by embryonic exposure.

There are few lasting effects of early cocaine exposure on overall longitudinal health of zebrafish as evidenced by the consistency in survival, sex ratio, body length or weight of the different longitudinal groups. Despite an obvious effect on telencephalic area at 5 dpf, no difference was detected in telencephalic area of longitudinal adults (Table 2), although the cellular architecture awaits description. This is in contrast to lasting deficits in cortical neurons reported for rodents and primates prenatally exposed to cocaine [11,16]. Despite the deleterious effects shown in mammalian model animals, there is still considerable debate on the clinical relevance because of the generally low toxicity of the drug reported in humans by other studies [4]. Zebrafish, with their poikilothermic capacity for regeneration (recently reviewed in [46]) might also be able to recover more completely from a developmental insult. That said, subtle, long-lasting effects on cardiovascular physiology and behavior were observed in zebrafish treated with cocaine during development.

While cocaine certainly changed heart rate in 5 dpf embryos, there were no longitudinal effects seen on baseline heart rate or rhythm in adults. Previous studies showed that acute treatment with cocaine elevates heart rate in a bell-shaped dose-response manner, with a maximal effect of 12% at 10 mg/L [29]. In the present study, three days of cocaine treatment increased heart rate in the same bell-shaped dose response manner, but the maximal increase (37%) was much higher than that seen previously with acute treatment. This increase was seen 24 h after the drug was removed, suggesting that the sympathetic tone of the fish was altered by cocaine treatment. As longitudinal adults, however, baseline heart rate of fish embryonically pre-exposed to cocaine was no different than untreated controls, suggesting that some form of compensation occurred. The lower response to isoproterenol suggests that ß1-adrenergic signaling, which mediates much of the effects of cocaine on the heart in all species examined, was attenuated in response to early exposure (Figure 4A). Similar data have been reported for neonatal rats treated with cocaine *in utero* in which prenatal cocaine altered ß-adrenergic receptor expression, as well as downstream regulatory genes of the signaling pathway [47,48]. The cardiovascular response to cocaine of adult fish from the embryonic treatment groups was not significantly different than untreated controls (*p* < 0.06). However, the relationship of acute adult response and embryonic exposure dose is bell-shaped, with a maximum response by fish pre-exposed to 10 mg/L. This is very similar to the dose response seen for the other heart parameters in embryos and adults. Together these data suggest strongly that the sympathetic outflow pathway is sensitized by developmental exposure. The zebrafish heart begins responding to adrenergic agonists at 4 dpf [49]. It is possible that longer embryonic exposure might have generated a more substantial longitudinal sensitization. We are currently exploring this possibility.

Similar sensitization is also evident in cocaine-induced CPP. Significant CPP using 5 mg/L cocaine was induced in fish from all embryonic treatment groups, except those treated with 20 mg/L. Once again, the dose response curve for the embryonic treatment groups was bell-shaped, with maximal effect at 10 mg/L. The response to cocaine in these fish was also significantly higher than the response of fish untreated as embryos. This suggests that early exposure sensitizes pathways regulating cocaine reward. Several studies have looked at development of the dopaminergic, noradrenergic and serotonergic systems in the zebrafish [42,50]. Tyrosine hydroxylase, the rate limiting enzyme in dopamine and noradrenergic synthesis is first expressed in cells of the ventral diencephalon at 18–22 hpf, which begin extending axons rostrally and caudally soon after. By 3 dpf, most of the major tracts have been established, including the likely teleost equivalent of the mesolimbic dopaminergic reward pathway characterized in mammals [24]. By five days, the overall pattern of cells and axon tracts is believed to be essentially equivalent to that in the adult. Indeed, at 5 dpf, the behavior of the embryo is more motivational in nature, including the pursuit and choice of food, and the possible beginning of social behavior [51,52]. However, true conditioned learning has not been demonstrated in zebrafish larvae until sometime later (reviewed in [53]). The window of treatment used in the present study must be important for reward sensitization, since CPP is significantly impacted in fish treated with 10 mg/L cocaine. Previous studies in zebrafish have shown the longitudinal effects of ethanol exposure on social behavior using this treatment window [54]. We are currently exploring the impact of longer exposure times during development.

The bell-shaped dose response curves seen in cocaine-induced tachycardia (CIT) and CPP for the different embryonic treatment groups suggest that pre-exposure makes the fish more sensitive to the drug as adults. In previous studies [27,32], we have shown that the acute dose response curve for both CPP and CIT is bell-shaped. In the case of CIT, the cause is likely due to cocaine’s effects on ion channels at higher concentrations [35,36]. The mechanism driving the U-shaped curve in zebrafish CPP has been noted by others working with rodents, and involves a combination of primary signaling pathway desensitization at higher doses and activation of less sensitive antagonistic pathways [55]. The GFP^+^ strain of fish used in this study were noticeably less sensitive to cocaine than fish we have used in the past. In previous studies, 5 mg/L cocaine typically increased preference approximately 12.5%–15% [27] and heart rate approximately 26% [29]. In the present study, CPP was only increased by 10% (Figure 5) and heart rate by 13% (Table 2 and Figure 4, respectively] in drug naïve fish (the 0 controls). Embryonically pretreated fish showed a progressive increase in both CIT and CPP, indicating that they became more sensitive to the drug. One possible explanation for why the fish pretreated with 20 mg/L showed a decreased response is that they were actually supersensitive and the 5 mg/L acute adult treatment was actually on the right side of the dose response curve. The reward pathways in rodents have been shown to have similar sensitization resulting from pre-natal exposure to cocaine. Mice and rats treated with cocaine between E7 and term showed altered vulnerability to develop cocaine self-administration [17]. Interestingly, other studies have shown that CPP is negatively impacted by embryonic exposure, but this could be due to a shift in the dose response curve relative to controls [56].

The connection between cardiovascular response and CPP is particularly intriguing because perhaps it represents a diagnostic tool for determining addiction vulnerability. In our previous work, we have seen a maximal effect on heart rate at 5 mg/mL [29] and a maximal effect on CPP at 10 mg/mL [27,28], suggesting that the cardiovascular response is more sensitive than the behavioral response. It has been reported in humans that the maximal subjective feeling of euphoria occurs at a lower dose than the maximal cardiovascular response [57]. It is not certain how euphoria (the subjective high feeling) corresponds with reward-based learning and addiction, but these results would suggest that the maximal cardiovascular effects of cocaine are aversive. Indeed, it has been reported that the tachycardia effect of cocaine negatively impacts the activity of reward circuits in rodents [58]. Very few studies have been done correlating cardiovascular response to behavioral response in model organisms. One study looking at cocaine-induced acute locomotor effects roughly corresponded with cardiovascular response in different strains of mice [59]. These locomotor effects are now known to reflect the rewarding effects on behavior in these strains [60]. In the present study, sensitization to the cardiovascular and behavioral effects correlated in a dose-specific fashion, suggesting that the sensitivity of the two pathways is inextricably linked. The genomic changes associated with both aspects of sensitization are also likely to be linked and are the subject of future research.

## 4. Materials and Methods

### 4.1. Fish Maintenance

All zebrafish for this study were housed in the facility at the University of North Dakota according to standard Institutional Animal Care and Use Committee recommendations (Animal Welfare Assurance #A3917-01, protocol 1403-7). Fish were raised in stand-alone fish racks (Aquatic Habitats, Apopka, FL, USA). Fish were fed twice daily, artemia in the morning and pellet in the afternoon. They were kept on a constant light-dark cycle: 14 h on, 10 h off. The line of fish used for these studies was a reporter line and was a generous gift [61]. This line expresses green fluorescent protein (GFP) driven by an α1-tubulin promoter that confines expression to the central nervous system (CNS). The GFP expression facilitated vital measurement of embryonic brain size. Fish heterozygous for the reporter gene were crossed to generate parallel lines of homozygous GFP-positive (GFP^+^) and homozygous GFP-negative (GFP^−^) fish. Imaging of embryonic fish and longitudinal adults was done on the homozygous GFP+ strain. The GFP^−^ line was used for experiments assessing cell death with acridine orange (AO).

### 4.2. Embryonic Drug Treatment

The basic experimental design in this study is diagrammed in Figure 1A. Homozygous GFP^+^ or GFP^−^ fish were bred pairwise overnight and the eggs collected the following morning. Embryos of a single clutch from one pairwise mating were staged to approximately 22–24 hours post fertilization (hpf) the following day (d1 in Figure 1A) [62]. Twenty-five to thirty embryos from the single clutch were transferred to each well of a 6 well plate with fish water (reverse osmosis water with 0.3 g/L Instant Ocean (Instant Ocean Spectrum Brands, Blacksburg, VA, USA) and 0.1 g/L sodium bicarbonate) or fish water with dissolved cocaine. Preliminary experiments showed that opening of the chorion did not affect the impact of cocaine exposure; thus, for these studies, the chorions were left intact. Cocaine (Sigma-Aldrich, St. Louis, MO, USA) was dissolved in fish water to different final concentrations (0, 2.5, 5, 10, and 20 mg/L). Typically, two concentrations were tested with a 0 control in each experiment and each condition was tested at least 6 times. Water supplemented with fresh drug was exchanged on days 2 and 3 (d2 and d3 in Figure 1A). The percentage of hatched embryos was recorded on day 2. On day 4 (d4 in Figure 1A), the fish were rinsed three times and given fish water without drug. The fish were imaged and tested for heart rate on day 5 (d5 in Figure 1A). Alternatively, fish from all five treatment conditions were grown to adulthood for longitudinal studies. In two experiments, the same fish imaged as embryos were then grown up and included in the longitudinal studies. For convenience, however, the other longitudinal experiments reported herein were conducted in parallel with fish used for imaging.

### 4.3. Imaging and Measuring Size and Brain Morphology

Imaging was done on day 5 using a Leica dissecting microscope (M165FC) (Heerbrugg, Switzerland), camera (DFC310FX) (Heerbrugg, Switzerland), and image analysis software suite (Leica Application Suite version 4.1.0, Leica Microsystems, Heerbrugg, Switzerland). Ten fish from each condition were immobilized by transferring to 0.2% methylcellulose in fish water. The fish were then imaged individually, belly down, under bright field illumination at low magnification (30–32×) to determine fish eye size and body length using Leica analysis software (see Figure 1A). The fish were then imaged at high magnification (120×) under fluorescence illumination to visualize the CNS by GFP expression. The image was later analyzed with Leica software to determine the area of fluorescence as an indicator of brain size. Three brain regions were defined for which the area was measured as shown in Figure 1B. The telencephalon included the region between the olfactory epithelium and the telencephalic flexure. For convenience in reporting, what we called diencephalon included the optic tectum, midbrain and cerebellum, and what we called the hindbrain included the rhombencephalon between the cerebellum and the constriction marking the spinal cord. Brains imaged all showed oval profiles for the olfactory epithelium, indicating full extension of the forebrain. The telencephalons of longitudinal adult fish brains were also imaged for GFP^+^ fish (not shown), but the skull and pigmentation prevented effective imaging of the other brain regions.

Acridine orange (AO) staining was used to determine if cocaine increased cell death in the forebrains of GFP^−^ embryonic zebrafish. The technique has been described by others [63]; however, briefly, after the same drug treatment described above, 5-day-old embryos were incubated in 1 µg/mL AO (Sigma-Aldrich) dissolved in fish water for 10 min, rinsed three times, and then imaged using a GFP filter at 120X. A depth of focus was chosen such that the surface pigment and olfactory epithelium were clearly in focus, then all visible AO^+^ cells were counted. The heart was also imaged to quantify AO positive cells (AO^+^), although very few were detected after cocaine treatment.

### 4.4. Measuring Embryonic Heart Rate

Embryonic heart rate of cocaine-exposed animals was measured as described previously [29]. Heart rate was measured on day 5, 24 h after cocaine cessation, to separate acute effects of the drug from longer lasting effects driven by three-day exposure. After imaging for size and brain morphology, embryos were turned on their sides, and allowed to recuperate for 5 min. A 12 s video at low resolution and maximal frame rate (69 frame/s) was shot of the beating heart imaged at high magnification (120×). The videos were then played back in slow motion and the number of beats was counted to determine embryonic heart rate. Counts from any one treatment group were performed by at least three investigators, at least one of whom was masked to treatment condition. In previous studies [29], we found that extensive manipulation of the larvae increased heart rate, so imaging was done from multiple angles. While heart rate was readily measureable from all angles, subtleties in heart morphology were difficult to assess. However, investigators did look for location of the ventricle and obvious arrhythmias in the larvae.

### 4.5. Conditioned Place Preference

Cocaine-induced conditioned place preference (CPP) was measured essentially as described previously [27,28]. Briefly, fish treated with various doses of cocaine as larvae were grown up at a density of 25–30 fish/3 L tank (see schematic in Figure 1A). At approximately 8 months of age, they were tested for cocaine-induced CPP. Eight months was an empirical threshold determined in previous studies by quantifying the number of fish that froze and thus failed to perform in the CPP test. Fish were removed from their home tank and housed individually during testing, which took 6 days. On day 1, the fish habituated to the CPP chamber during a 45 min exposure. The CPP apparatus, described previously [28], was divided into three sections with perforated walls that allowed fish access to all sections and which could be replaced with solid walls for confinement during drug exposure. On day 2, a baseline preference for each section was recorded during a 10 min trial. The fish were then confined in the front compartment for 30 min and then confined for 30 min in the back. On day 3, the same procedure was repeated except that the confinement order was reversed: fish were initially confined to the back and then later the front. On day 4, fish were tested for their final baseline, after which preference for the two smaller end compartments was calculated. The fish were then confined to the more preferred compartment without drugs for 30 min. They were then confined to the least preferred compartment for 30 min with cocaine (5 mg/L). On day 5, the first CPP was recorded and the conditioning paradigm determined on day 4 was repeated. On day 6, a final CPP was recorded. Percent change in preference was determined by subtracting the baseline preference for the least preferred compartment determined on day 3 from the average of the two CPP trials on days 5 and 6.

### 4.6. Electrocardiograms in Adult Zebrafish

Electrocardiograms (ECGs) were recorded as described previously [29]. Fish were sedated with 40 µg/L tricaine (MS-222, Sigma-Aldrich), placed on a damp sponge belly up and ventilated by a peristaltic pump that continuously perfused the gills and kept the animal sedated. A few ventral scales were removed and two 29-gauge microelectrodes (AD Instruments, Colorado Sprigs, CO, USA) inserted to a depth of approximately 1 mm, one in the thorax between the opercula and one in the abdomen, near the anal fins (the ground electrode was placed in the sponge nearby). Electrical signals detected by the electrodes were amplified and translated by a PowerLab data acquisition unit using LabChart 7.2.1 software (AD instruments, Colorado Springs, CO, USA). Recordings were made in the range of 0–10 mV, and digital filters limiting frequency range to 8–40 Hz were applied for analysis. Once a signal was detected, the pump was turned off to decrease noise and the ECG was recorded for 1 min. The pump was turned on immediately thereafter, but the water source was changed to include water without cocaine, or water with either 5 mg/L cocaine, or 20 µM isoproterenol. These doses provided optimal heart rate increases in previous experiments [29]. After 1 min of drug administration, the pump was turned off and a second recording taken. Average heart rate was determined by counting the number of QRS complexes per minute (the QRS complex denotes ventricular depolarization and is the most obvious wave form in the ECG trace). All baseline ECG traces were examined for evidence of arrhythmia. Tricaine was used as an anesthetic, and we have found that this caused some irregularity in heart rate for most of the ECG traces. However, it was possible in five traces to detect a pattern of extra P-waves (the P-wave denotes atrial depolarization), or skipped beats within a stretch of otherwise normal rhythm.

## 5. Statistics

Comparisons between treatment groups were done using one-way ANOVA (Prism 5.0, GraphPad Software Inc., LaJolla, CA, USA). Bonferroni’s post-test was used to determine pairwise differences, or Dunnett’s post-test to compare experimental groups to untreated controls. In order to combine data from several brain morphology experiments, the brain region size of cocaine-treated fish in a given experiment was expressed as area (Table 1) or expressed as a percentage of untreated fish from the same experiment (Figure 2C). ANOVA was run after compilation of results from several experiments with the number indicated in the legend. When percentage of control was used for comparison, values were transformed to arcsin for statistical comparison. Thus, the *p*-values shown in Figure 2C were from transformed data even though the data is plotted as percentages of control values. Similarly, the hatching and survival data were expressed as percentages in Table 1 but were transformed for statistical analysis to determine *p*-values. For CPP measurements, repeated-measure ANOVA was used to verify CPP for individual longitudinal treatment groups. One-way ANOVA with Bonferroni’s post-test was then used to assess differences between longitudinal treatment groups.

## 6. Conclusions

We have shown that early embryonic exposure in zebrafish sensitizes their behavioral and physiological sensitivity to the drug later in adulthood. In many respects the action of cocaine on zebrafish development is similar to that reported in other model organisms. Interestingly, embryonic exposure dose is the key to sensitization later. The nature of how the system is sensitized is not yet known, but judging from early effects on the telencephalon, it is quite possible that the brain cytoarchitecture underlying, or impacting, reward-based learning and sympathetic output is in some way disrupted. Future studies looking at regional markers will test this hypothesis. Alternatively, the expression of critical gene networks underlying reward and sympathetic output might be modified by early exposure. Future transcriptomic studies will address this possibility. If these gene expression patterns are indeed modified, the question of possible transgenerational inheritance of these modifications immediately arises. Such a prospect would be very exciting in light of recent evidence for epigenetic inheritance of addiction-related behaviors in rodents [64].

## Figures and Tables

**Table 1:** Morphometry of embryonic zebrafish clutches treated with cocaine. Larval fish treated with different doses of cocaine were inspected for percentage of hatched individuals at 48 h post fertilization (hpf) and then imaged for size analysis at 5 days post fertilization (dpf) One-way ANOVA with Dunnett’s post-test was used to compare embryonic treatment groups to the untreated control fish. For percentage hatched, values were arcsin transformed before statistical evaluation, *p* < 0.0001, *F* = 7.69, df = 47, * *p* < 0.05, ** *p* < 0.01, and *** *p* < 0.001 when compared to untreated fish. For body size *p* < 0.9651, *F* = 0.145, and df = 214. For embryonic eye diameter (size), *p* < 0.123, *F* = 1.834, and df = 221. For telencephalic (Tel.) area *p* < 0.015, *F* = 3.24, df = 110, * *p* < 0.05 when compared to untreated fish. For the diencephalon (Dien.) area, *p* < 0.145, *F* = 1.745, and df = 122. For hind brain (Hind.), *p* < 0.149, *F* = 1.727, and df = 114. For acridine orange positive (AO^+^) cells in the telencephalon, *p* < 0.891, *F* = 0.279, and df = 109. For AO^+^ cells in the diencephalon, *p* < 0.113, *F* = 1.92, and df = 109.

Embryonic Treatment Condition	0 mg/L Cocaine	2.5 mg/L Cocaine	5.0 mg/L Cocaine	10.0 mg/L Cocaine	20.0 mg/L Cocaine
% Hatched by 48 hpf	16 ± 15.5	39.5 ± 21.3	51.5 ± 13.8 *	59.1 ± 19.5 **	65.5 ± 28.9 ***
Body size (mm)	3.97 ± 0.16	3.94 ± 0.15	3.96 ± 0.19	3.95 ± 0.21	3.95 ± 0.13
Eye Size (mm)	0.352 ± 0.016	0.344 ± 0.014	0.351 ± 0.016	0.346 ± 0.023	0.351 ± 0.16
Tel. Area (mm^2^)	0.0306 ± .0028	0.0294 ± 0.0026	0.0292 ± 0.0026	0.0283 ± 0.0025 *	0.0280 ± 0.0035 *
Dien. Area (mm^2^)	0.117 ± 0.009	0.111 ± 0.006	0.112 ± 0.010	0.111 ± 0.011	0.113 ± 0.013
Hind. Area (mm^2^)	0.0499 ± 0.0060	0.0503 ± 0.0058	0.0495 ± 0.0057	0.0463 ± 0.0035	0.0494 ± 0.0049
AO^+^ Tel AO^+^ Dien	6.41 ± 0.97 5.27 ± 1.34	6.96 ± 1.09 5.68 ± 0.91	7.5 ± 0.87 9.00 ± 0.87	6.55 ± 0.83 7.57 ± 1.51	6.36 ± 0.73 6.64 ± 0.69

**Table 2:** Overall health of longitudinal adults treated with cocaine as embryos. Fish treated with one-way ANOVA with Dunnett’s post-test was used to compare embryonic treatment groups to the untreated control fish. For percentage survival at 8–10 months, *p* < 0.98, *F* = 0.080, df = 28. For the percentage of males, *p* < 0.55, *F* = 0.7748, df = 34. Fish were classified as either male or female, even in cases where the sex was ambiguous therefore the percentage of females can be inferred from this data as well. For body length (Len.), *p* < 0.205, *F* = 1.52, and df = 132. For body weight (Wt.), *p* < 0.420, *F* = 0.98, df = 132. For telencephalic area (Tel. Area), *p* < 0.16, *F* = 1.55, df = 84. For Baseline Heart Rate (HR), *p* < 0.7704, *F* = 0.4522, df = 60.

Embryonic Treatment Condition	0 mg/L Cocaine	2.5 mg/L Cocaine	5.0 mg/L Cocaine	10.0 mg/L Cocaine	20.0 mg/L Cocaine
Survival at 8–10 months	61.5% ± 7.8	63.5% ± 7.4	62.7% ± 7.6	63.2% ± 13.2	66.8% ± 11.2
Percentage of Males	34.9% ± 14.1	50.3% ± 8.7	55.2% ± 11.0	43.8% ± 12.9	62.5% ± 9.2
Body Len. (cm)	3.6 ± 0.1	3.8 ± 0.3	3.7 ± 0.2	3.8 ± 0.2	3.6 ± 0.2
Body Wt. (g)	0.45 ± 0.07	0.44 ± 0.10	0.48 ± 0.10	0.43 ± 0.09	0.44 ± 0.13
Tel. Area (mm^2^)	1.86 ± 0.21	1.98 ± 0.22	1.97 ± 0.3	2.09 ± 0.27	2.11 ± 0.38
Baseline HR	138.3 ± 9.5	144.5 ± 8.0	132.9 ± 6.2	138.6 ± 6.5	130.5 ± 9.8
